# The family business: turning sleep into dreams

**DOI:** 10.1093/sleepadvances/zpad036

**Published:** 2023-12-27

**Authors:** Anthony Kales, Joyce D Kales, Helen C Kales

**Affiliations:** Department of Psychiatry, Pennsylvania State University School of Medicine, Hershey, PA, USA; Department of Psychiatry, Pennsylvania State University School of Medicine, Hershey, PA, USA; Department of Psychiatry, University of California Davis School of Medicine, Sacramento, CA, USA

## Childhood

First and foremost, I am the son of Greek immigrants, Demetrios and Demetra (Partalis) Kales from the tiny mountainous, Grecophone village of Kato Lesnitsa in Northern Epirus directly on the Greek-Albanian border. Kato Lesnitsa was poor and war-torn, occupied by the Italian army during my parents’ childhoods during World War I and other repeated conflicts. My parents experienced extreme hardship and realized that they should emigrate to the United States (my father in the 1920s and my mother in the 1930s) to better themselves. They were prescient because, during World War II, Greece would be invaded by Italy, occupied by the Nazis, and then subject to a brutal civil war with a focal point of conflict being their village and culminating with the village being annexed by then Stalinist Albania. The extreme adversity experienced by my relatives who were left behind the Iron Curtain pushed me to make a difference later in life for them when I could.

**Figure 1.**
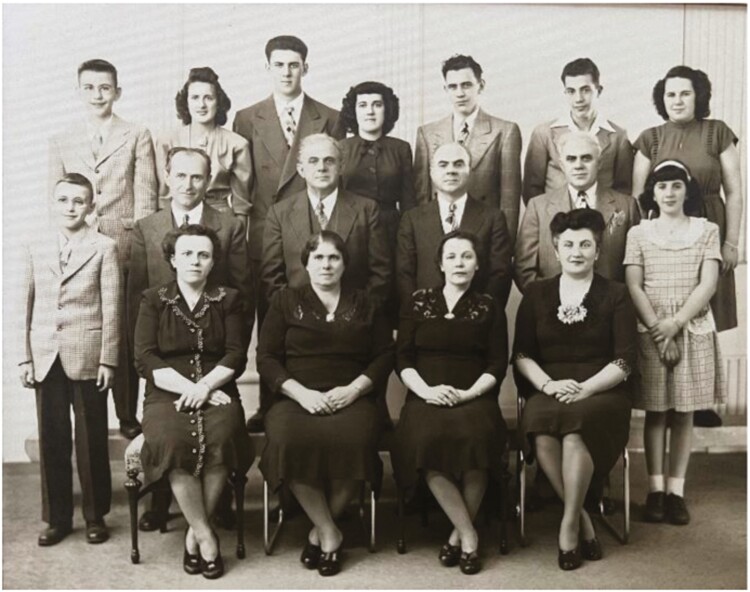
Extended Greek Family circa 1944. Dr. Anthony Kales, bottom left.

Together with their extended family (Figure 1) and starting in the area of Detroit known as “Greektown,” my father, his brother, and his brothers-in-law owned a series of small businesses. Despite their difficult circumstances and limited education (my mother only completed the sixth grade), my parents were extremely resilient in the face of setbacks including during the Depression. If one business failed, they would start another. Over time, this included six businesses: a shoe-shine parlor, confectionary, two luncheonettes, and two taverns. My father became seriously ill when I was a child, and my mother worked tirelessly in the family bar to help support the family.

## Education

**Figure 2.**
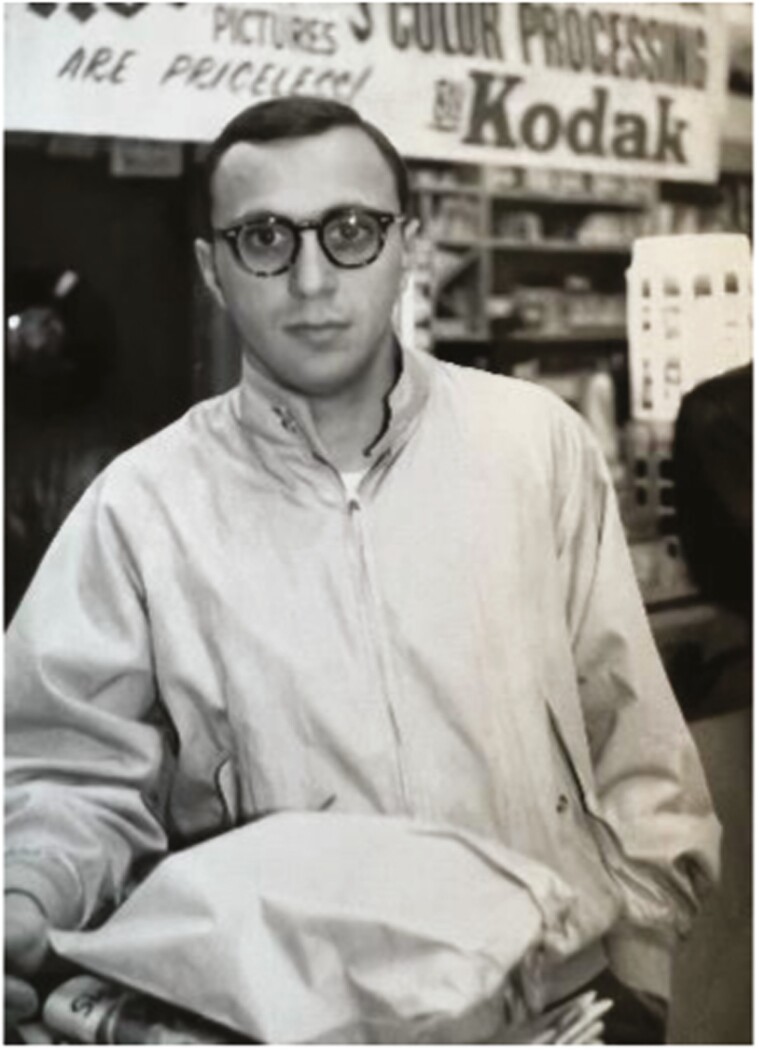
Dr. Anthony Kales was a teenager 1950s.

English was my second language, and I was eager for educational role models. Heavily influenced by the Greek family physician, Dr. Vassilios Moisides, who cared for the Greektown community including my father, I became the first person in my family to go to college (Figure 2). The only affordable option I had was to attend college locally at Wayne State University (WSU), working at Stroh’s Brewery and the Post Office in order to pay for classes and help my family. I graduated in 1956 with high distinction and as a member of Phi Beta Kappa Honor Society.

**Figure 3.**
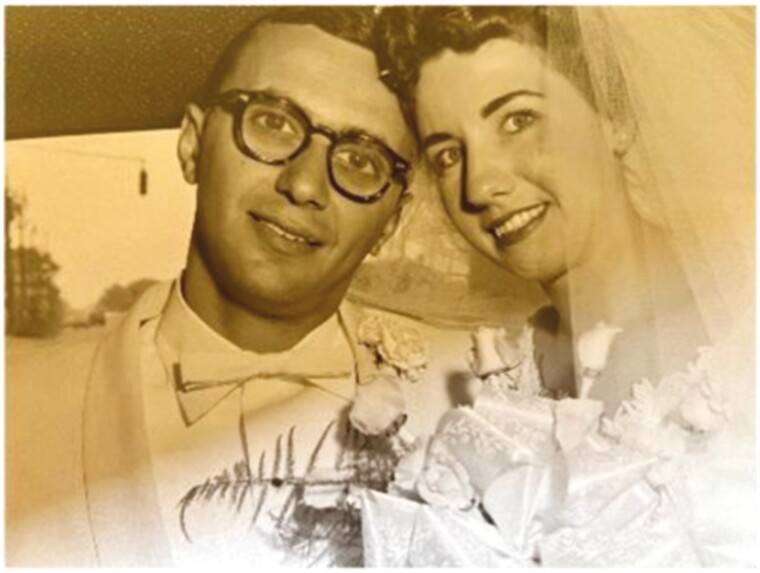
Drs. Anthony and Joyce Kales, Wedding 1960.

While at WSU in 1952, I met the woman who would become my partner in work and in life, Joyce Danielski Kales. Joyce, a pharmacy student, and I met when we were matched up as lab partners during an undergraduate biology frog experiment. After college, I was accepted into the WSU School of Medicine while Joyce completed her studies in the School of Pharmacy. We dated for seven years. Our families were initially opposed to a marriage as we came from different religious and ethnic backgrounds (Greek Orthodox and Polish Catholic). During this time, I convinced Joyce to go on to medical school (at the time, there were very few women in medicine) and our families to accept our relationship. We were married in separate Greek Orthodox and Catholic ceremonies in 1960 (Figure 3).

During medical school, I had become captivated by what made people “tick” and realized that in Psychiatry, one had the biggest opportunity to change people’s lives and improve their functioning. While psychiatry at the time was steeped in psychoanalysis (which fascinated me), I also believed from this early stage in the links between the biological, the psychological, and the social. I did my first research project as a senior medical student on the “Psychological Effects of Hysterectomy” for which I received the American Medical Association Student Research Award.

After graduation from WSU in 1959 (and later induction into the Alpha Omega Alpha), I traveled to Los Angeles to enter a psychiatry residency at UCLA and the famed Neuropsychiatric Institute. I chose UCLA for its strength in both psychotherapy and nascent biological psychiatry. In addition, Los Angeles of the 1960s was an incredibly exciting place; for the son of immigrants, with limited exposure to different experiences and other cultures, I was avid in learning new pursuits including theater, classical music, traveling, dining experiences, collecting art, and running. Joyce followed in 1960, pursuing a psychiatry residency as well. I completed my Psychiatry Residency in 1963 and joined the faculty at UCLA where I became a full Professor in 1971.

## Introduction to Sleep Research

While at UCLA, I became fascinated by the science of sleep after the discovery of Rapid Eye Movement (REM) sleep by Aserinsky and Kleitman [1]. Specifically, I realized the centrality of sleep to most, if not all, psychiatric disorders. With fellow senior resident Frederick (Fritz) Hoedemaker as part of our concomitant Master of Science degrees, we conducted a dream deprivation experiment using ourselves as participants; we each took several nights, awakening each other every time a REM period was indicated by EEG. We found that there was an increase in the number of awakenings needed to prevent dreaming and an increase in dream time percentage on the first recovery night, substantiating the hypothesis of a “need to dream.” This work was later published in *Nature* [2]

Following graduation, I entered private practice in Westwood, and while successful, I continued to pursue sleep research. In 1962, I returned to the academic environment at the UCLA Neuropsychiatric Institute, and founded the Sleep Research and Treatment Center. This Center was the first such clinically integrated research program in the United States to systematically evaluate normal sleep across various age groups, as well as all known sleep disorders including: insomnia, sleepwalking, night terrors, nightmares, enuresis, narcolepsy/cataplexy and sleep apnea.

**Figure 4.**
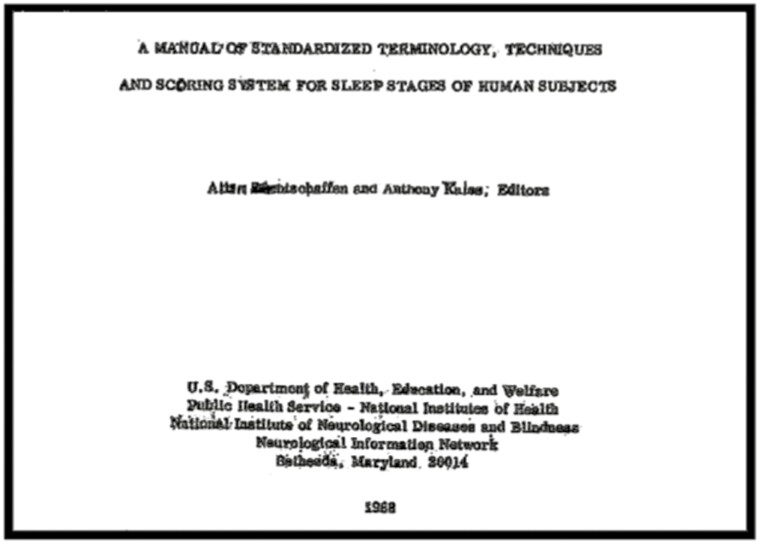
Rechtschaffen and Kales Sleep Scoring Manual.

Allan Rechtschaffen and I understood that to become a science, sleep research would require a standardized terminology and method of recording the stages of sleep. In 1968, Allan and I would co-edit *A Manual of Standardized Terminology, Techniques, and Scoring System for Sleep Stages of Human Participants (*Figure 4) [3]. We convened a panel of experts to agree to a standardized method of the scoring of sleep stages, which were divided into wakefulness, stages 1–4 (non-REM), and REM. At least one EEG lead was recommended (C3 or C4 referenced to the opposite ear or mastoid), as well as two electrooculogram (EOG) leads and a submental electromyography (EMG) lead. The method we devised recommended dividing the polysomnographic record of sleep into 30-second epochs, beginning at the start of the sleep study.

We did not know it at the time, but this manual would become the standard for the field, and for 40 years was the most widely referenced publication in sleep research. This comprehensive training manual served as the foundation for both Modern Sleep Research as well as the new subspecialty of Sleep Disorders Medicine. This is because this training system initiated and maintained standardized sleep stage scoring, not only in studies of normal sleep, but also in delineating sleep stage abnormalities with and between sleep disorders. The strength and enduring value of this manual were attested to by the fact that for four decades since its publication, scientists were convened with the express purpose of modifying its various standards and components—however, these convocations did not result in any significant changes to the training program/manual until 2007.

## Transition to Pennsylvania State University, Founding Chair of Psychiatry

In 1971, I decided that to solidify and integrate my career goals across the missions of medicine, I could best achieve them by becoming a Chair of Psychiatry. By this time, Joyce and I had three children and I sought a position where our academic pursuits could come to fruition along with a place that would be ideal to raise our children. We moved the family and the Sleep Research and Treatment Center to the Pennsylvania State University College of Medicine (Hershey, PA), one of the very few new medical schools at the time. I became the founding Chair of Psychiatry at the age of 37 years. Despite this heavy administrative lift, I was determined to continue to pursue my research.

## Key Findings from the Sleep Research and Treatment Center

In addition to myself and my wife, Dr. Edward Bixler left UCLA to join the PSU Sleep Research and Treatment Center. The Center would eventually attract faculty and international collaborators including Drs. Constantine Soldatos, Antonio Vela-Bueno, and Alexandros Vgontzas. Together we would delineate and correlate the clinical characteristics, course, demographic factors, and sleep laboratory findings for each sleep disorder. Our fundamental starting point was a comprehensive sleep history and psychological testing, far more definitive in most sleep disorders than routine use of all-night polyhypnographic studies.

Some of our many original findings are summarized as follows:

- *Insomnia* [4–11].
  ◦ Most prevalent sleep disorder with high levels of morbidity and distress.
  ◦ Increases in frequency with age, among women with stress, and mental health disorders.
  ◦ The severity of sleep disturbance is strongly related to the degree of underlying/associated psychopathology.
  ◦ Those with insomnia have characteristic personality patterns, most predominantly anxious-depressive, especially dysthymic depression.
  ◦ Emotional arousal is very common, with rumination, anxiety, and depression, this emotional arousal leads to physiological hyperarousal and sleep difficulty (hyperarousal mechanism for the development of insomnia).
  ◦ Physiological hyperarousal is strongly reinforced and maintained by conditioning factors (fear of sleeplessness and performance anxiety)—mechanism for the perpetuation of insomnia.
  ◦ The disorder of insomnia is a 24-hour disorder of hyperarousal with important implications for its proper diagnosis and treatment.
- *Parasomnias* [12–16].
  ◦ Sleepwalking and night terror events are not the acting out of a dream, but occur out of nonrapid eye movement (NREM) sleep, especially stages 3 and 4 (deep sleep).
  ◦ Nightmares occur out of REM or dreaming sleep.
  ◦ Night terrors and nightmares can be easily differentiated from one another in the clinical laboratory.
  ◦ Sleepwalking and night terrors in childhood are usually secondary to developmental factors, whereas these disorders in adults are usually related to psychopathology.
- *Disorders of Excessive Daytime Sleepiness* [17–21].
  ◦ Narcoleptic patients with cataplexy require sleep laboratory evaluation.
  ◦ For sleep apnea, laboratory evaluation should occur when apnea is suggested by the sleep or partner history.
  ◦ The symptoms and signs of sleep apnea (snoring, excessive daytime sleepiness, nocturnal snorting, and gasping sounds, as well as observer-noted nocturnal breathing cessations) generally become manifest before the age of 40 years.
  ◦ Those with hypertension are at high risk for sleep apnea with the most severe sleep apnea associated with the highest blood pressure levels. In men, sleep apnea peaks at the age of 55 years, whereas in women, peaks at the age of 65 years.
  ◦ Obesity without sleep apnea is associated with daytime sleepiness which is a result of circadian abnormalities rather than just being secondary to nighttime sleep disturbances.
- *Evaluation of Psychoactive Drugs in the Sleep Laboratory* [22–56].
  ◦ We pioneered the use of the sleep laboratory to evaluate the efficacy of hypnotics and other psychoactive drugs. This original work led to a number of important accomplishments and original findings.
  ◦ I was the primary author of the FDA’s Guidelines for Evaluating Hypnotic Drugs, which were developed after I introduced the sleep laboratory as the primary tool for assessing hypnotic drugs and their effectiveness.
  ◦ The original findings in the sleep laboratory related to the delineation of important mechanisms and syndromes and consequent side effects and adverse effects of short- and long-acting hypnotic drugs and high-potency benzodiazepines, including:
    ▪ With short half-life benzodiazepines, we identified their mechanisms and consequent adverse effects, including rebound insomnia and early morning insomnia.
    ▪ With high-potency benzodiazepines, we delineated their mechanisms and associated adverse effects of memory impairment and other cognitive impairments.
    ▪ With long half-life benzodiazepines, we identified adverse effects of carryover daytime sedation.
    ▪ We correlated these side effects and adverse effects with pharmacokinetics (half-lives) and pharmacodynamics (potency) of the respective hypnotic drugs, leading to a number of important changes by the FDA in drug labeling and dosage, as well as changes in physicians’ prescribing patterns.

## Scientific Impact of Research and Publications and Recognitions

Over my career, I have authored or co-authored 310 scientific articles and book chapters, as well as six books. The high scientific impact of our research group’s publications is indicated by the extraordinarily high number of times they have been cited in the literature. Moreover, A Manual of Standardized Terminology; Techniques and Scoring System for Sleep Stages of Human Subjects was for 4 decades the most highly cited publication of the sleep research field.

The scientific impact of our group’s publications is reflected in the exceptional Scientific Citation Index, but also in the high quality of the journals in which they frequently appear. For example, in the following 10 highly prestigious journals, we have published a total of 69 articles: American Journal of Psychiatry (9), Annals of Internal Medicine (7), Archives of Internal Medicine (3), Clinical Pharmacology and Therapeutics (11), Journal of the American Medical Association (4), JAMA Psychiatry (9), Journal of Clinical Endocrinology (10), Lancet (6), New England Journal of Medicine (4), and Science (6). It is important to emphasize that these studies published by our group in the most prestigious journals collectively represent the only comprehensive body of research from one sleep research center across all of the major sleep disorders including insomnia, narcolepsy/cataplexy, sleep apnea, and the parasomnias (sleepwalking, night terrors, and nightmares). Importantly, these studies have produced much information on the psychosocial and behavioral mechanisms associated with each disorder and their implications for evaluation and treatment. This, in turn, has provided researchers and clinicians with a substrate for each of these disorders that has fostered the expansion of research and clinical activity in Sleep Disorders Medicine. In 2003, in recognition of my body of work, I was honored to be recognized as one of the Founders of Modern Sleep Research. In addition, I was bestowed the WSU Distinguished Alumnus Award, the Hellenic Medical Society Distinguished Physician Award, and an Honorary Doctorate from the University of Athens.

## Mentoring and Development of the “Next Generation”

I have had the honor and opportunity to mentor and train many outstanding medical students and postdoctoral research fellows in the field of Sleep Disorders Medicine and Psychiatry. In addition to their training and collaborative research with our group in the Sleep Research and Treatment Center, five of these scientists have gone on to establish highly successful independent and internationally renowned research careers in Sleep Disorders Medicine and Psychiatry. These distinguished scientists include:

- Constantin R. Soldatos MD: Dr. Soldatos was Chairman, Department of Psychiatry, President, Union of Professors of Medicine, and Director Sleep Research Unit, all at University of Athens Medical School. In addition, he was President of both the Hellenic Society of Academic Psychiatry and the Hellenic Society for the Advancement of Psychiatry and Related Sciences. He is the major founder of the field of Sleep Disorders Medicine in Greece, and is internationally recognized for his scientific contributions toward the importance of Psychiatry within the overall field of Sleep Disorders Medicine.
- Edward O. Bixler PhD: Dr. Bixler was Vice Chair for Research and Co-Director of the Sleep Research and Treatment Center in the Department of Psychiatry at the Pennsylvania State University College of Medicine. He is internationally recognized for his pioneering research in the Epidemiology of Sleep Disorders, particularly insomnia and sleep apnea, with an emphasis on the effects of age, sex, and behavioral/psychiatric factors.
- Antonio Vela-Bueno MD: Dr. Vela Bueno was a Professor, Director of the Sleep Laboratory, and Director of the Stress and Insomnia Research Center, all at the Autonomous University, Madrid, Spain. He was the major founder of Sleep Disorders Medicine in Spain and was the founder and first President of the Iberian Association of Sleep Pathology. He has established annual, international symposia on Stress, Sleep, and Insomnia. Dr. Vela Bueno is internationally recognized for his contributions to the field of Stress and Insomnia, particularly as they relate to the workplace environment and occupational medicine.
- Alexandros N. Vgontzas MD: Dr. Vgontzas is currently the Director of the Sleep Research and Treatment Center at the Pennsylvania State University College of Medicine. He is internationally recognized for his pioneering research in the Neuroendocrinology and Neuroimmunology of Sleep Disorders, especially of Sleep Apnea and Insomnia with a special emphasis on the underlying pathophysiologic mechanisms of these disorders. Dr. Vgontzas has pioneered the novel concept that sleep apnea is a manifestation of metabolic syndrome and objective sleep time is useful in phenotyping insomnia. For his contribution to sleep science, he has been awarded the Anthony Kales Endowed Chair in Sleep Disorders Medicine. Finally, Dr. Vgontzas has served as Chair and Professor of the Department of Psychiatry at the University of Crete-Greece with special emphasis on the development of community psychiatry services in underserved rural areas of Crete.
- Dennis Charney MD: He was Dean of Research, Dean for Academic and Scientific Affairs, and is the Anne and Joel Ehrenkranz Dean at Icahn School of Medicine at Mount Sinai in New York, and Executive Vice President for Academic Affairs at the Mount Sinai Health Center. Prior to his tenure at Mount Sinai, he was Chief of the Mood and Anxiety Research Program, Experimental Therapeutics and Pathophysiology Branch of the National Institute of Mental Health and Professor of Psychiatry at Yale University. He is internationally recognized for his research work in Psychiatry particularly in neurobiology and treatment of mood and anxiety disorders. He is currently President of Academic Affairs at the Mount Sinai Health System.

## The Family Business

As with so many other immigrants, Greek families like mine helped each other to succeed in their new homeland. Growing up, we lived in a duplex next to my mother’s brother and sister-in-law and hosted numerous cousins who came over from Greece as they established themselves. Our businesses were shared within the family including “Pete’s Bar” in Detroit, which was a partnership between my Uncle (Theo) Pandelis (Pete) and my parents. When members of my extended family were able to leave Stalinist Albania in the 1990s, I helped each of them to become established in Greece or in the United States. In this spirit, when I took on faculty or mentees including numerous medical students, they became a part of the family, and I would try to give them the same support and “start” that a family member would receive.

**Figure 5.**
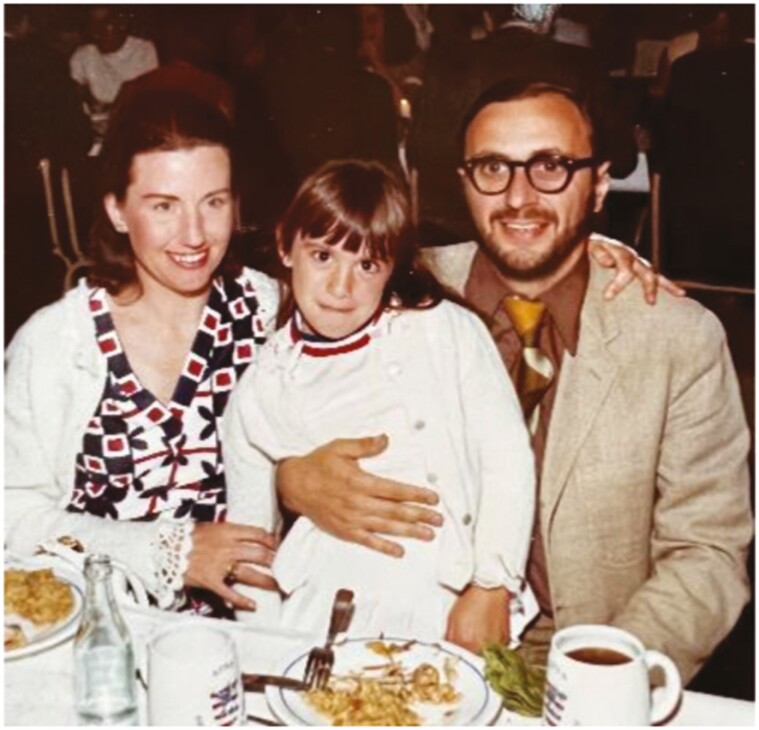
Dr. Helen Kales (age 6) with Drs. Anthony and Joyce Kales, Association for the Psychophysiological Study of Sleep (APSS), Bruges Belgium, 1971.

**Figure 6.**
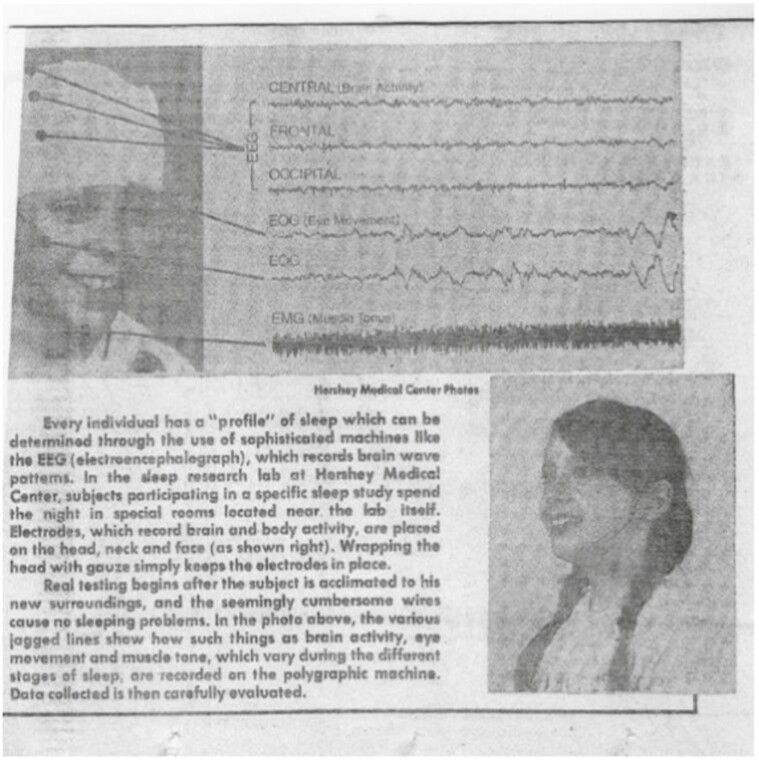
Drs. Stefanos (age 9) and Helen (age 7) Kales in sleep training videos, 1972.

Additionally, just as I had worked in my parent’s business, I exposed our own children Stefanos (born 1963), Helen (Eleni; born 1965), and Jim (Demetrios; born 1967) to research from an early age. As we traveled to scientific conferences, we took the children with us, often taking them out of school, reasoning that what they would learn from visiting other places and countries was invaluable (Figure 5). When they were young, they even participated in training videos for sleep practitioners (Figure 6).

When they became teenagers, they each worked in the research hub of the Department known as the “Data Lab.” I am proud that for each child, it spurred an intellectual curiosity that has manifested in each of their careers and in turn, in our seven grandchildren (Anastasia, Sophia, Alexandra, Stefania, Zoe, Theodoros, and Arianna).

## Philanthropy: Giving Back

I consider myself to be incredibly fortunate to have been able to create a wonderful personal and professional life for myself with the support of so many family members and mentors. The motto Phi Beta Kappa (Philosophia Biou Kivernitis, translated from Greek as love of wisdom governs life) has been a major guiding principle for me and my wife.

We believe that education is the path forward given our experiences from humble beginnings. In this regard, we have initiated, established, and fundraised major endowment funds to provide yearly scholarships to high school seniors at the three Greek Orthodox church communities that we have attended, with over 200 scholarships awarded to date. We have also made substantial commitments to support in an endowed manner, major clinical, educational, and research programs at our alma maters, WSU, and WSU School of Medicine.

Lastly, we are committed to the revitalization of Detroit; in addition to our support of Wayne State, we have been patrons of the Detroit Symphony Orchestra and the Hellenic Museum of Michigan. I was honored to receive the Hellenic Heritage Award in 2009 for my dedication to the advancement of the Greek-American community and broader community at large in the Greater Detroit area.

## Notes

This paper is part of the Living Legends in Sleep Research series, which is sponsored by Idorsia Pharmaceuticals and Jazz Pharmaceuticals.

Living Legends: Dr. Anthony Kales MD MS
